# Associations between physical activity expectations, health literacy, and life satisfaction in cancer patients: results of a quantitative cross-sectional study

**DOI:** 10.1007/s00432-026-06653-z

**Published:** 2026-10-27

**Authors:** Franziska Ostendorf, Emilia Hintze, Luise Schütze, Christian Keinki, Dorota Lubgan, Ludwig Fischer von Weikersthal, Christoph Stoll, Gianluca Ciarlo, Jens Büntzel, Jutta Hübner

**Affiliations:** 1 Department of Internal Medicine II, Hematology and Medical Oncology, Comprehensive Cancer Center Central Germany, University Hospital Jena, Jena, Germany; 2 Department of Radiation Oncology, University Hospital Erlangen, Erlangen, Germany; 3 Department of Hematology and Medical Oncology, St. Marien Hospital Amberg, Amberg, Germany; 4 Department of Internal Medicine and Hematology, Herzoghöhe Hospital, Bayreuth, Germany; 5 Medical Clinic I, University Hospital, Goethe University Frankfurt, Frankfurt am Main, Germany; 6 Department of Otorhinolaryngology, South Harz Hospital Nordhausen, Nordhausen, Germany

**Keywords:** Cancer patients, Health literacy, Physical activity, Complementary and alternative medicine (CAM), Life satisfaction, Integrative oncology

## Abstract

**Purpose:**

Health literacy, physical activity, and complementary and alternative medicine (CAM) are increasingly recognized as important factors influencing coping strategies, quality of life, and well-being in cancer patients. However, empirical evidence on their interrelationships remains limited.

**Methods:**

We conducted a multicenter cross-sectional study between April and November 2024 at one Comprehensive Cancer Center and eleven oncological sites across Germany. A total of 316 cancer patients completed a standardized questionnaire assessing health literacy, self-reported sport participation, expectations toward physical activity, affective attitudes toward physical activity, CAM use, spirituality, nutrition, and life satisfaction. Descriptive statistics, reliability analyses, t-tests, and Spearman’s correlations were performed.

**Results:**

Among the 316 participants, health literacy (M = 3.10, SD = 0.44) and life satisfaction (M = 7.03, SD = 2.02) were high. Health literacy did not differ significantly by self-reported pre-diagnosis sport participation. Life satisfaction likewise did not differ by self-reported pre-diagnosis sport participation. In contrast, more positive affective attitudes toward physical activity were significantly correlated with health-related expectations toward physical activity (*r* = 0.580, *p* < 0.001), body-related motives (*r* = 0.471, *p* < 0.001), and life satisfaction (*r* = 0.212, *p* < 0.001). CAM use was frequent, with 44.9% reporting vitamin D supplementation and 37.5% other vitamins or dietary supplements.

**Conclusion:**

While self-reported sport participation did not show significant associations with life satisfaction, motivational and cognitive factors—such as positive attitudes and expectations—were linked to health literacy and well-being. These findings highlight the importance of strengthening patient-centered communication, motivational counseling, and health literacy as key elements of integrative oncology.

## Introduction

A cancer diagnosis can represent a profound psychological and emotional challenge and may be accompanied by shock, fear, helplessness, and uncertainty (Antoni et al. 2023; Krieger et al. 2023). Coping with the new life situation is significantly influenced by patients’ health literacy(Zhang et al. 2023).Life satisfaction can be understood as a cognitive, global evaluation of one’s life and is a core component of subjective well-being (Diener et al. 1999).

Health literacy refers to the ability of individuals to find, understand, and apply health-related information to make informed decisions that positively impact their health in both the short and long term. This ability is particularly important as it enables patients not only to process relevant health information but also to make targeted, evidence-based decisions regarding the prevention, promotion, and maintenance of their health. Health literacy includes various dimensions that are essential for finding, understanding, and applying health-relevant information (Sørensen et al. 2012).Functional health literacy refers to basic skills such as reading, writing, and accessing health information, while interactive health literacy describes the ability to evaluate and apply this information. Critical health literacy, in turn, includes the social and cognitive skills to question acquired information and place it in a broader context (Nutbeam 2008).The overarching goal of these competencies is to sustainably improve personal quality of life by making informed decisions that positively affect individual health.

Limited health literacy is common in oncology and can hinder patients’ ability to navigate complex care pathways and to actively participate in decisions (Harding et al. 2022). Difficulties in finding and evaluating reliable health information have also been described in cancer populations (Le et al. 2022).In line with established models that differentiate functional, communicative, and critical health literacy, these competencies are linked to patients’ participation in the health-care process (Ishikawa and Yano 2008a; Ishikawa et al. 2008b).

In addition to established conventional medical treatments such as chemotherapy, radiation therapy, classical surgery, or radiotherapy, complementary medical approaches are increasingly being integrated into treatment. Complementary and alternative medicine (CAM) encompasses a wide range of non-conventional medical practices that are used as supplements or alternatives to conventional medicine (National Cancer Institute 2024). These complementary methods, such as acupuncture, herbal medicine, or physical activity like yoga, have the potential to enhance patients’ quality of life and can contribute to better coping with the disease by supporting both physical and psychological aspects of recovery. CAM is often used to relieve symptoms and improve well-being (Lin et al. 2019). Integrative medicine (IM) refers to the targeted combination of conventional medical procedures with evidence-based complementary approaches. It places particular emphasis on patient-centered care that addresses not only the treatment of the disease but also the physical, emotional, and social needs of patients (Witt et al. 2017).It includes non-pharmacological therapeutic approaches that have traditionally been used outside of modern Western medicine but have shown positive effects for patients in recent research. When systematically integrated into standard cancer care, these therapeutic forms can complement conventional treatment approaches and improve the quality of care for cancer patients.

The aim of this study was to investigate how selected components of integrative oncology—particularly physical activity, health literacy, and the use of complementary and alternative medicine (CAM)—are associated with life satisfaction in cancer patients. In doing so, we focused not only on the prevalence of CAM use and the level of health literacy, but especially on how motivational and cognitive factors—such as expectations toward physical activity and perceived health-related competence—relate to overall life satisfaction. This approach shifts the focus from merely behavioral indicators to underlying attitudes and psychosocial correlates.

## Methods

The study was designed as a prospective multicenter cross-sectional study and was conducted at the Comprehensive Cancer Center Jena and eleven cooperating oncological institutions across Germany. These included the Klinik Herzoghöhe in Bayreuth (Department of Hematology and Oncology), the Oncological Competence Center at Klinik Bad Trissl, the Oncological Forum Celle e.V. (Cancer Counseling Center and Outpatient Palliative Care Service), the MVZ Onkologische Kooperation Harz in Goslar, the Department of Radiation Oncology at University Hospital Erlangen, the Breast Center of St. Barbara Klinik Hamm-Hessen, the Städtisches Krankenhaus Kiel GmbH, the Integrative Oncology Day Clinic of Klinikum Südstadt Rostock, the Department of ENT, Head and Neck Surgery, Interdisciplinary Palliative Care Unit, and Sleep Laboratory at Südharz Klinikum Nordhausen, the Klinikum St. Marien in Amberg, the Joint Practice for Radiology and Radiation Therapy at St. Josefs Hospital, and the Department of Hematology and Internal Oncology at Jena University Hospital. Data collection took place between April 1, 2024, and November 30, 2024. In this article, we present data on the influence of health literacy, physical activity, and CAM on the physical well-being of cancer patients. We included all patients with a diagnosis of cancer and sufficient proficiency in the German language to complete the questionnaire independently. The questionnaire was distributed in printed form by the staff of the participating centers.

### Participants

A total of 316 cancer patients were recruited from multiple study sites. At the Comprehensive Cancer Center Jena and eleven cooperating oncological institutions across Germany, questionnaires were distributed either in person by the research team or by collaborating healthcare professionals. Patients were asked to complete the questionnaire anonymously and by hand. Inclusion criteria were age > 18, a confirmed cancer diagnosis, ongoing oncological treatment, and sufficient German language proficiency to independently complete the questionnaire.

### Questionnaire

In this study, a standardized questionnaire was used, comprising six thematically structured sections: (1) demographic data, (2) questions on health literacy (Seck et al. 2026), (3) items related to complementary and alternative medicine (CAM), (4) questions on physical activity (Brehm et al. 2010), (5) questions addressing spirituality and life satisfaction (Peterman et al. 2002), and (6) questions concerning dietary habits (nutritionDay worldwide 2018).

For this purpose, an anonymized, standardized questionnaire was developed to assess health-related competencies, information behavior, physical activity, CAM use, and general life satisfaction.

The questionnaire was developed to gain a comprehensive understanding of health-related behaviors, including those pertaining to physical activity, complementary and alternative medicine, nutrition, and spirituality, in individuals diagnosed with cancer.

*Part 1* collected demographic and disease-related information, including age, gender, education, socioeconomic status, tumor type, time of initial diagnosis, and current or previous treatment modalities. The items were formulated as multiple-choice questions with additional free-text fields for individualized responses.

*Part 2* assessed health literacy using 11 items addressing participants’ handling of health-related and medical information. The items were organized into four domains: finding information (3 items), understanding information (3 items), appraising information (2 items), and applying information/acting on information (3 items). Responses were recorded on a four-point response scale (always, often, rarely, never), with higher scores representing greater self-reported health literacy. An overall health literacy score was calculated as the mean of 10 items. Internal consistency of the scale was evaluated using Cronbach’s alpha (Seck et al. 2026).

*Part 3* addressed the use of complementary and alternative medicine (CAM). This section consisted of ten dichotomous questions (yes/no) concerning specific methods (e.g., acupuncture, homeopathy, mistletoe therapy, meditation) as well as an optional open-ended question for individual comments (Hübner et al. 2014a, b, c).

*Part 4* focused on physical activity-related constructs and distinguished three aspects: self-reported sport participation, expectations toward physical activity, and affective attitudes toward physical activity.

The first five items assessed self-reported sport participation before diagnosis and at the time of the survey (yes/no), preferred types of sport, awareness of exercise groups for cancer patients, and the desire to engage in more sport. In the analyses, “physically active” and “inactive” refer to participants’ yes/no self-report of engaging in sport, rather than to an objective assessment of physical activity volume, intensity, or guideline adherence.

This was followed by 18 statements assessing expectations toward regular physical activity. Items were rated on a seven-point Likert-type scale ranging from “does not apply at all” to “fully applies” and were summarized into four expectation domains:

- Body-related motives (e.g., fitness, resilience, body shape),
- Positive health (e.g., stress reduction, well-being, experiencing nature),
- Social experience (e.g., belonging, social contacts, group activity),
- Performance/presentation (e.g., competition, self-image) (Brehm et al. 2010).

**Fig. 1:**
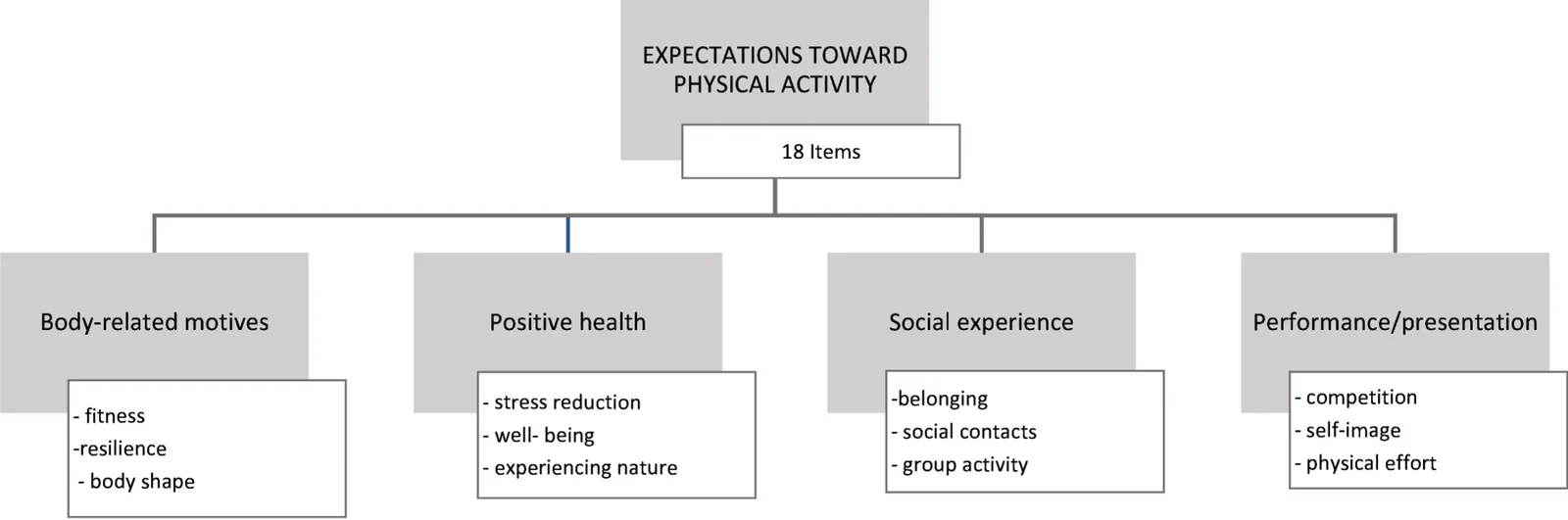
Conceptual grouping of the 18 items assessing expectations toward physical activity into four expectation domains

The conceptual grouping of the 18 expectation items into these four domains is shown in Fig. 1. In addition, four semantic-differential items assessed affective attitudes toward the idea of being physically active on seven-point scales (not relaxed–extremely relaxed, not satisfied–extremely satisfied, not happy–extremely happy, and not comfortable–extremely comfortable) (Brehm et al. 2010).

*Part 5* captured life satisfaction and spirituality. Items on spirituality addressed personal beliefs, existential and religious questions, and spiritual resources. Life satisfaction was assessed using Likert-type scales and a metric rating (1–10) (Peterman et al. 2002).

*Part 6* focused on dietary habits during the course of illness. In addition to general information on dietary behavior (multiple-choice and yes/no questions), 11 items measured the frequency of food consumption using a scale ranging from never/seldom to ≥ 6 times per day. Additional questions addressed eating behavior in the past few days (nutritionDay worldwide 2018).

### Statistical analysis

Statistical analyses were performed using IBM SPSS Statistics, Version 29.0.2.0. To characterize the sample and key variables—such as health literacy, life satisfaction, and self-reported sport participation—descriptive statistics (means, standard deviations, skewness) were computed. To assess expectations toward physical activity, the 18-item scale described above was applied (Brehm et al. 2010). Descriptive values for each expectation subscale (body-related motives, positive health, social experience, performance/presentation) were also calculated.

To evaluate the internal consistency of the applied multi-item scales, Cronbach’s alpha was computed, with values ≥ 0.70 considered acceptable(Tavakol and Dennick 2011). Item analyses were conducted, including corrected item-total correlations and Cronbach’s alpha if item deleted. Group comparisons for health literacy and life satisfaction according to self-reported sport participation were performed using independent-samples t-tests. Homogeneity of variances was assessed using Levene’s test (Levene 1960). To examine associations between affective attitudes toward physical activity, expectations toward physical activity, health literacy, life satisfaction, and duration of treatment, Spearman’s rank correlation coefficient (Spearman’s rho) was calculated.

This non-parametric method was chosen for ordinal or non-normally distributed data (Schober et al. 2018). Correlations were interpreted as small (|ρ| = 0.10–0.29), moderate (|ρ| = 0.30–0.49), or strong (|ρ| ≥ 0.50), following Cohen’s conventional benchmarks (Cohen 1988). Statistical significance was set at *p* < 0.05; correlations with *p* < 0.01 were additionally denoted by **.

### Ethics approval and consent to participate

Participation in the survey was voluntary and anonymous; informed consent was implied by returning the completed questionnaire. The study was approved by the Ethics Committee of the University Hospital Jena, Germany, on March 28, 2024 (Approval No. 2024-3307-Bef).

## Results

### Demographic data

A total of 316 patients participated in the study. Among the participants, 203 (64.2%) were female and 109 (34.5%) were male. The average age of the participants was 61.8 years (SD = 11.8). Participants’ ages ranged from 23 to 85 years. The most common cancer types reported in the sample were breast cancer (34.5%), colon cancer (14.2%), digestive tract cancer (12.3%), and lung cancer (10.8%). Other frequent diagnoses included gynecological cancers (7.9%) and prostate cancer (4.1%). In addition, 16.8% of patients reported a variety of other tumor types (e.g., skin cancer, lymphoma, multiple myeloma, ovarian cancer, pharyngeal cancer, etc.; see Table 1).

**Table 1:** Sociodemographic and clinical characteristics of the study population

	*N* = 316
Age in years; mean ± sd (min-max)^a^	61.84 ± 11.77 (23–85)
*Gender n* (%)	
Female	203 (64.2)
Male	109 (34.5)
No answer	4 (1.3)
*Marital status n* (%)	
Married	203 (64.2)
Divorced	36 (11.4)
Widowed	35 (11.1)
Single	33 (10.4)
No answer	9 (2.9)
*Religion n* (%)	
Protestant	93 (29.4)
Catholic	79 (25.0)
Atheistic	34 (10.8)
Orthodox	4 (1.3)
Muslim	2 (0.6)
Jewish	1 (0.3)
other	8 (2.5)
No answer	97 (30.7)
*Level of education n* (%)	
Middle school (until 10th grade)	118 (37.3)
Middle school (until 9th grade)	77 (24.4)
High school graduation (until 12th /13th grade)	57 (18.0)
University degree	47 (14.9)
No graduation	1 (0.3)
No answer	16 (5.1)
*Type of cancer n* (%)	
Breast cancer	109 (34.5)
Colon cancer	45 (14.2)
Digestive tract cancer*	39 (12.3)
Lung cancer	34 (10.8)
Gynaecological cancer	25 (7.9)
Prostate cancer	13 (4.1)
Urinary tract cancer	5 (1.6)
Other**	53 (16.8)
No answer	4 (1.3)
Time since diagnosis in years; mean ± sd (min-max)^b^	2.82 ± 4.13 (0–29)
*Current treatment n* (%)^c^	
Chemotherapy	159 (50.3)
Operation	70 (22.2)
Other medication	63 (19.9)
Radio therapy	51 (16.1)
Hormones	31 (9.8)
Other***	28 (8.9)

^a^*n* = 289; ^b^*n* = 303; ^c^Multiple answers possible. n.d.= not disclosed. sd = standard deviation. min = minimum. max = maximum

*Including: stomach cancer, esophageal cancer, pancreatic cancer, cancer of the bile ducts or liver

**Other cancer types (*n* = 53): skin cancer (*n* = 3), lymphoma (*n* = 4), multiple myeloma (*n* = 1), bone marrow cancer (*n* = 1), ovarian cancer (*n* = 2), liver cancer (*n* = 1), lymph node cancer (*n* = 2), tonsil cancer (*n* = 1), pharyngeal cancer (*n* = 1), pancreatic cancer (*n* = 1), metastases (*n* = 3); the remaining participants did not specify the cancer type

***Other treatments (*n* = 28): antibody therapy (*n* = 1), brachytherapy (*n* = 2), follow-up care (*n* = 1), tamoxifen (*n* = 1); the remaining participants did not specify the treatment received

### Health literacy

The internal consistency of the 11-item scale assessing comprehension, decision-making and application of medical information was high, with a Cronbach’s alpha of α = 0.86. Corrected item-total correlations were mostly within the acceptable to good range (*r* = 0.454– 0.596). One exception was the item “How often do you find the information you are looking for when seeking information about treatment options for your illness?”, which showed a markedly lower item discrimination with a correlation of *r* = 0.111.

The average score on the overall health literacy scale was M = 3.10 (SD = 0.44), indicating that participants generally reported a high level of health literacy. The distribution of scores was approximately normal (skewness = – 0.002, SE = 0.137), suggesting a homogeneous pattern of responses without significant skewness.

A comparison of Cronbach’s alpha values with hypothetical item exclusion showed that omitting individual items had only a minor impact on the internal consistency of the scale.

### Physical activity

To assess the internal consistency of a scale measuring affective responses to the idea of engaging in physical activity, an item analysis was conducted. Corrected item-total correlations ranged from *r* = 0.864 to *r* = 0.921, indicating consistently high values and strong conceptual coherence among the items. Cronbach’s alpha values upon hypothetical item exclusion ranged from α = 0.938 to α = 0.955, suggesting that removing any single item would not improve internal consistency. Overall, the scale demonstrated excellent internal consistency and reliability (total Cronbach’s alpha estimated > 0.95).

Participants’ affective attitudes toward physical activity (*N* = 285) showed a mean of M = 4.94 (SD = 1.56) on a seven-point scale. Like the distribution observed for health literacy, a negative skewness (– 0.769, SE = 0.144) indicated a generally positive affective attitude toward physical activity.

In the domain of expectations related to physical activity, four subscales were evaluated. The highest mean values were found for the subscales “Body-related motives” (M = 5.05, SD = 1.40; *N* = 297) and “Positive health” (M = 4.76, SD = 1.34; *N* = 298). In contrast, the subscales “Social experience” (M = 3.84, SD = 1.51; *N* = 288) and “Presentation/performance” (M = 2.61, SD = 1.47; *N* = 288) showed comparatively lower means. The skewness of these distributions ranged from nearly symmetrical (e.g., social experience: – 0.009) to pronounced positive skew (e.g., Presentation: 0.897), indicating less agreement with sport-related motives linked to self-presentation.

### Life satisfaction

A total of 305 patients responded to the question regarding their overall life satisfaction. Life satisfaction was measured on a scale from 1 to 10, yielding a mean score of 7.03 (SD = 2.02). The distribution was negatively skewed (skewness = − 1.025), indicating that many participants reported high levels of life satisfaction. This suggests that, on average, cancer patients in this sample were relatively satisfied with their lives. The slightly positive kurtosis (0.788) suggests a relatively peaked distribution, with values more strongly clustered around the mean.

### Complementary and alternative medicine

Table 2 presents the percentage of participants using CAM methods. Out of a total of 312 participants, 44.9% (*n* = 140) reported using “Vitamin D,” making it the most frequently mentioned CAM intervention. The second most common category was “Other vitamins, dietary supplements, or minerals,” reported by 37.5% (*n* = 117) of the patients. Usage data for other CAM methods varied widely. A detailed overview of the percentage distributions and absolute frequencies is provided in the following table.

**Table 2:** Use of complementary and alternative medicine (CAM)

	*N* = 312
*Vitamin D*	
Yes	140 (44.9)
No	172 (55.1)
*Other vitamins*,* food supplements*,* or minerals*	
Yes	117 (37.5)
No	195 (62.5)
*Medicinal plants*,* medicinal teas or secondary plant substances (e.g. Chinese or Ayurvedic herbs/teas*,* curcumin*,* green tea extracts)*	
Yes	48 (15.4)
No	264 (84.6)
*Mistletoe*	
Yes	5 (1.6)
No	306 (98.4)
*Probiotics*	
Yes	9 (2.9)
No	303 (97.1)
*Detoxifying and cleansing procedures*	
Yes	14 (4.5)
No	298 (95.5)
*Special diets (e.g. ketogenic diet*,* vegan diet*,* fasting)*	
Yes	20 (6.4)
No	291 (93.3)
*Acupuncture/acupressure*	
Yes	9 (2.9)
No	302 (96.8)
*Yoga/Tai Chi/Qi Gong*	
Yes	28 (9.0)
No	284 (91.0)
*Laying on of hands or spiritual healing*	
Yes	3 (1.0)
No	308 (98.7)
*Homeopathy*	
Yes	24 (7.7)
No	287 (92.3)

### Associations between health literacy, physical activity, and life satisfaction in cancer patients

To explore whether health literacy may be associated with self-reported sport participation prior to cancer diagnosis—and whether this is reflected in differences in life satisfaction—descriptive group comparisons were conducted. Participants who reported sport participation before their cancer diagnosis showed comparable levels of health literacy (M = 3.10, SD = 0.42, *n* = 170) compared to those who reported no sport participation before diagnosis (M = 3.10, SD = 0.46, *n* = 138). Similarly, only minimal differences were observed in life satisfaction: participants reporting pre-diagnosis sport participation reported a mean score of M = 7.03 (SD = 1.91), while participants reporting no pre-diagnosis sport participation reported a mean of M = 6.99 (SD = 2.15). These results suggest no substantial group differences in health literacy or life satisfaction. Independent samples t-tests confirmed the absence of statistically significant differences in both health literacy (t(306) = 0.006, *p* = 0.995) and life satisfaction (t(299) = 0.204, *p* = 0.838). Levene’s tests indicated homogeneity of variances (health literacy: *p* = 0.342; life satisfaction: *p* = 0.109). These findings do not suggest a clear relationship between self-reported pre-diagnosis sport participation and health literacy or life satisfaction.

### Health literacy and life satisfaction

Participants reported similar levels of health literacy regardless of whether they had reported sport participation prior to their cancer diagnosis (M = 3.10) or not. Likewise, participants reporting current sport participation (M = 3.14) and participants reporting no current sport participation (M = 3.08) showed only minimal differences. A comparable pattern emerged for overall life satisfaction, with mean values ranging from M = 6.90 to M = 7.17 across all subgroups. Additionally, no significant difference in life satisfaction was found between participants who expressed a desire to engage in more sport and those who did not.

### Expectations toward physical activity

Clear differences emerged in the subscales assessing expectations toward physical activity:

#### Body-related expectations

Participants who reported sport participation prior to their cancer diagnosis, those who reported current sport participation, and those who expressed a desire to engage in more sport attributed significantly greater personal benefit to exercise in terms of fitness, physical appearance, resilience, and body awareness (e.g., “I improve my fitness,” “I get a more toned body”). In all three groups, mean values exceeded M = 5.4, whereas lower values were observed in the respective comparison groups (e.g., M = 4.52–4.70), indicating that physical activity is associated with stronger body-related motivational expectations in participants reporting sport participation or a desire to engage in more sport.

#### Health-related expectations

A similar pattern was observed in the subscale assessing health-related expectations toward physical activity, which included aspects such as stress reduction, general well-being, and experiencing nature.

Participants who reported sport participation before their cancer diagnosis, those who reported current sport participation, and those who expressed a desire to engage in more sport reported significantly higher values on this subscale (M = 5.18–5.34) compared to participants who reported no sport participation or did not express such a desire (M = 4.20–4.32). These findings suggest that participants reporting sport participation or a desire to engage in more sport perceive stronger health-promoting benefits of engaging in physical activity.

#### Social experience

Participants reporting sport participation also had higher expectations regarding the social effects of physical activity (e.g., social interaction, group experience). While this difference was statistically significant across all subgroups, it was somewhat less pronounced (current sport participation: M = 4.09; pre-diagnosis sport participation: M = 4.02 vs. no sport participation: M = 3.50–3.63). To further examine the relationships between core study variables—namely affective attitudes toward physical activity, health literacy, life satisfaction, treatment duration, and expectations toward physical activity— Spearman’s rank-order correlations were calculated. Affective attitudes toward physical activity showed significant correlations with all four expectation subscales: Body-related (*r* = 0.471, *p* < 0.001), Health (*r* = 0.580, *p* < 0.001), Appearance/Performance (*r* = 0.242, *p* < 0.001), and Social Experience (*r* = 0.328, *p* < 0.001). Additionally, a significant relationship was observed between affective attitudes toward physical activity and health literacy (*r* = 0.142, *p* = 0.016) as well as life satisfaction (*r* = 0.212, *p* < 0.001). However, the previously reported lack of group differences in life satisfaction based on the desire to engage in more sport (see group comparisons above) suggests that affective attitudes toward physical activity may be a more sensitive indicator of psychological benefit than expressed behavioral intentions alone. Health literacy was also significantly associated with Body-related expectations (*r* = 0.230, *p* < 0.001) and Health-related expectations (*r* = 0.216, *p* < 0.001), but not with Appearance (*r* = 0.015, n.s.) or Social Experience (*r* = 0.085, n.s.). Life satisfaction correlated positively with both health literacy (*r* = 0.189, *p* = 0.001) and Body-related expectations (*r* = 0.142, *p* = 0.016), indicating a modest link between psychological well-being and functional health knowledge as well as physical self-perception. Treatment duration was negatively associated with Body-related expectations (*r* = – 0.134, *p* = 0.023), Health expectations (*r* = – 0.137, *p* = 0.020), and Appearance (*r* = – 0.137, *p* = 0.020), but not with Social Experience (*r* = – 0.063, n.s.).

## Discussion

The present study examined how self-reported sport participation, expectations and affective attitudes toward physical activity, health literacy, and life satisfaction were related in cancer patients. No significant differences in health literacy or life satisfaction were observed according to self-reported pre-diagnosis sport participation. In contrast, affective attitudes toward physical activity were associated with expectations toward physical activity and showed small positive correlations with health literacy and life satisfaction. These findings support a differentiated discussion of behavioral, cognitive, and motivational aspects without implying causal relationships.

### Health literacy in the context of the study sample

In this study, participants reported comparatively high levels of self-reported health literacy, and neither pre-diagnosis nor current self-reported sport participation was significantly associated with health literacy. Only weak correlations with affective attitudes toward physical activity were observed. However, these cross-sectional findings should not be interpreted as evidence that health literacy is a stable individual trait. Health literacy is better understood as a context-dependent construct whose skills and competencies can develop over the life course as contextual demands and experiences change (Sørensen et al. 2012). The comparatively favorable health literacy scores in our sample should be interpreted in the context of findings from the general population. In the second Health Literacy Survey Germany (HLS-GER 2), 58.8% of adults in Germany were classified as having low health literacy, with difficulties in appraising health information being particularly prevalent. Thus, the relatively high health literacy observed in our sample may not be representative of the broader German population (Schaeffer et al. 2021).

However, the findings cannot be directly compared, as HLS-GER 2 used a different instrument and scoring system. The relatively high health literacy observed in our sample may partly reflect selection effects associated with study participation, as participants may differ systematically from the general population. Participation was voluntary, participants were required to complete the questionnaire independently, and sufficient German language proficiency was an inclusion criterion. Individuals who were more confident in dealing with health information or more interested in health-related topics may therefore have been more likely to participate. This interpretation should be considered with caution, as voluntary participation may have resulted in a sample that was more health-conscious than the general population.

### Life satisfaction: multifactorial and independent of self-reported sport participation

Participants reported generally high levels of life satisfaction, and no significant differences were observed according to self-reported pre-diagnosis sport participation. This finding contrasts with literature reporting beneficial effects of physical activity and psychological outcomes and quality of life among cancer patients and survivors (Fong et al. 2012).

Evidence syntheses have highlighted benefits of exercise for cancer prevention and survivorship (McTiernan et al. 2019) and exercise interventions have been shown to improve health-related quality of life in cancer survivors in systematic reviews (Mishra et al^2012^). In addition, motivational strategies may support physical activity behavior in oncology settings (Takemura et al. 2024).

The difference between the present findings and intervention studies may partly reflect differences in how physical activity was assessed. In the present study, sport participation was captured by binary self-report questions, whereas structured exercise interventions assess planned and repeated activity. Fernández-Sánchez et al. (2024) concluded in a systemic review and meta-analysis that physical exercise programs may improve life satisfaction in people with or after cancer. These findings do not contradict the present results, but suggest that structured exercise interventions and a binary indicator of self-reported sport participation capture different aspects of physical activity (Fernández-Sánchez et al. 2024).

Psychological distress, including depression, is frequent in cancer and may influence disease trajectories and patient outcomes (Spiegel and Giese-Davis 2003).

Moreover, cancer patients may undergo a process of response shift, involving changes in internal standards, values, or the conceptualization of quality of life in response to changes in health status (Sprangers and Schwartz 1999; Ilie et al. 2019).

Changes in individual frames of reference may influence how experiences, including those associated with physical activity, are reflected in evaluations of life satisfaction.

In such cases, life satisfaction may be more strongly influenced by existential concerns, psychological adjustment, and psychosocial resources than by isolated health-promoting behaviors.

Empirical evidence supports this interpretation. Perceived social support has been associated with life satisfaction and quality of life among cancer patients (Faraci and Bottaro 2022). Similarly, the presence of meaning in life and spiritual resources has been linked to better psychological adjustment and higher well-being (Jim et al. 2006; Ano and Vasconcelles 2005).

Taken together, these findings suggest that psychosocial and existential factors may be important correlates of well-being and life satisfaction in patients with cancer. In the context of the present findings, such factors may therefore contribute to life satisfaction independently of self-reported sport participation.

### Expectations toward physical activity

The differences were particularly evident in expectation regarding physical activity. Patients who reported participated in sports, either before or after their diagnosis, or who expressed a desire to become more active reported significantly higher expectations in the domains of physical experience, health, and social interaction. High mean values were found in the “Health” subscale, underscoring the perceived role of physical activity in reducing stress and enhancing well-being. This interpretation is consistent with previous evidence demonstrating health-related benefits of physical activity in cancer survivors (Schmitz et al. 2010). Conversely, the lowest ratings were observed in the “Appearance” subscale, regardless of participants’ self-reported sport participation, suggesting that physical activity is primarily framed as a health-promoting rather than a performance- or image-oriented endeavor.

Positive correlations between affective attitudes toward physical activity and all four expectation domains support the relevance of these constructs: more favorable affective attitudes were associated with higher expectations, particularly for health-related and body-related outcomes. At the sample level, these associations were stronger than the correlations of affective attitudes with health literacy or life satisfaction. This pattern suggests that expectations and affective attitudes capture related motivational dimensions of physical activity, while their associations with broader outcomes such as life satisfaction remain comparatively small. The relevance of individual-level factors, including motivation, for physical activity behavior has also been described in previous research (Bauman et al. 2012).

### Limitations and future directions

This study has several limitations. Sport participation was assessed using binary self-report questions without considering type, intensity, or duration, which may have obscured relevant differences. The cross-sectional design precludes causal interpretation, and the use of self-report measures carries the risk of bias. Moreover, health literacy was captured in a restricted form of this multidimensional construct, and voluntary participation may have favored more health-conscious patients.

Despite these constraints, the study offers valuable insights into the associations between health literacy, physical activity, and life satisfaction in cancer patients and points to important directions for future longitudinal and multidimensional research.

### Clinical implications

The present findings support a patient-centered approach in which physical activity counseling considers not only whether patients report participating in sport, but also their expectations and affective attitudes toward physical activity. Because the observed associations with life satisfaction and health literacy were small and the study was cross-sectional, the findings should not be interpreted as evidence that changing these factors will directly improve well-being. Nevertheless, comprehensible and accessible health information, shared decision-making, and individualized discussion of expectations may help patients make informed choices about supportive care and physical activity.

## Conclusion

In this multicenter cross-sectional study of 316 cancer patients, self-reported pre-diagnosis sport participation was not associated with differences in health literacy or life satisfaction, whereas health literacy showed a weak positive association with life satisfaction. Affective attitudes toward physical activity were associated with several expectation domains, particularly health-related and body-related expectations, and showed a small positive association with life satisfaction. These findings highlight the importance of distinguishing self-reported sport participation from cognitive and affective aspects of physical activity when considering supportive care in patients with cancer. Given the cross-sectional design and potential selection of the study sample, the findings should be interpreted cautiously and do not allow causal conclusions. Longitudinal and intervention studies are needed to further examine the relationships between health literacy, physical activity-related expectations and attitudes, physical activity behavior, and life satisfaction.

## Data Availability

The datasets generated and analysed during the current study are available from the corresponding author on reasonable request.
